# Gut Microbiota Changes in Patients with Bipolar Depression

**DOI:** 10.1002/advs.201900752

**Published:** 2019-05-15

**Authors:** Shaohua Hu, Ang Li, Tingting Huang, Jianbo Lai, Jingjing Li, M. Elizabeth Sublette, Haifeng Lu, Qiaoqiao Lu, Yanli Du, Zhiying Hu, Chee H. Ng, Hua Zhang, Jing Lu, Tingting Mou, Shaojia Lu, Dandan Wang, Jinfeng Duan, Jianbo Hu, Manli Huang, Ning Wei, Weihua Zhou, Liemin Ruan, Ming D. Li, Yi Xu

**Affiliations:** ^1^ Department of Psychiatry First Affiliated Hospital Zhejiang University School of Medicine Hangzhou 310003 China; ^2^ The Key Laboratory of Mental Disorder's Management of Zhejiang Province No. 79, Qingchun Road Hangzhou 310003 China; ^3^ Brain Research Institute of Zhejiang University Hangzhou 310003 China; ^4^ Henan Gene Hospital The First Affiliated Hospital of Zhengzhou University Zhengzhou 450052 China; ^5^ Zhejiang University School of Medicine Hangzhou 310058 China; ^6^ State Key Laboratory for Diagnosis and Treatment of Infectious Diseases Collaborative Innovation Center for Diagnosis and Treatment of Infectious Diseases The First Affiliated Hospital School of Medicine Zhejiang University Hangzhou 310003 China; ^7^ Research Center for Air Pollution and Health Zhejiang University Hangzhou 310003 China; ^8^ Department of Psychiatry Columbia University New York NY 10032 USA; ^9^ Department of Obstetrics & Gynecology Hangzhou Red Cross Hospital Hangzhou 310003 China; ^10^ The Melbourne Clinic Department of Psychiatry University of Melbourne Melbourne Victoria 3052 Australia; ^11^ Department of Mental Health Ningbo First Hospital Ningbo 315010 China

**Keywords:** 16S rRNA gene sequence, biomarkers, bipolar disorder, gut microbiota, quetiapine

## Abstract

This study aims to characterize the gut microbiota in depressed patients with bipolar disorder (BD) compared with healthy controls (HCs), to examine the effects of quetiapine treatment on the microbiota, and to explore the potential of microbiota as a biomarker for BD diagnosis and treatment outcome. Analysis of 16S‐ribosomal RNA gene sequences reveals that gut microbial composition and diversity are significantly different between BD patients and HCs. Phylum *Bacteroidetes* and *Firmicutes* are the predominant bacterial communities in BD patients and HCs, respectively. Lower levels of butyrate‐producing bacteria are observed in untreated patients. Microbial composition changes following quetiapine treatment in BD patients. Notably, 30 microbial markers are identified on a random forest model and achieve an area under the curve (AUC) of 0.81 between untreated patients and HCs. Ten microbial markers are identified with the AUC of 0.93 between responder and nonresponder patients. This study characterizes the gut microbiota in BD and is the first to evaluate microbial changes following quetiapine monotherapy. Gut microbiota‐based biomarkers may be helpful in BD diagnosis and predicting treatment outcome, which need further validations.

## 1 Introduction

Bipolar disorder (BD), a chronic and recurrent disease, has a worldwide prevalence of around 0.8%.^1^, ^2^ BD is associated with severe impairment in cognitive and social functions,^2^ and increased risk of disability and suicide, especially in young individuals.^3^ To date, the pathogenesis of BD has not been fully elucidated. Interactions between genetic and environmental factors may play a role,^4^ along with biological alterations such as immune activation, metabolic disturbance, oxidative stress, and circadian rhythm abnormality.^5^

In recent years, the role of the brain–gut–microbiota axis in maintaining physical and mental well‐being has attracted accumulated attention. As a bidirectional modulation system, this axis builds a bridge between the brain and the gut through neuroanatomical, neuroimmune, and neuroendocrine pathways.^6^ Gut microbial alterations were observed in many diseases, including inflammatory bowel disease,^7^ autoimmune diseases,^8^ obesity,^9^ metabolic syndrome,^10^ and neuropsychiatric disorders.^11^ However, available evidence of gut microbiota in maintaining health is mostly obtained from animal studies, and relevant human studies are still in the infancy stage.

Hitherto only a few studies have preliminarily explored the gut microbiota in BD patients. Compared with healthy subjects, decreased abundance of *Faecalibacterium* in BD patients was observed, and shown to be associated with self‐reported symptoms and disease severity.^12^ Another study also reported decreased *Faecalibacterium* and *Ruminococcaceae* in BD patients.^13^ In this study, bacterial clades associated with inflammatory status, metabolic profiles, oxidative stress, and depressive symptoms have also been identified.^13^ BD patients treated with atypical antipsychotics showed altered composition of microbiota communities, especially in females.^14^ In patients with newly diagnosed BD, an association between genus *Flavonifractor* and BD was reported, but could be confounded by factors such as smoking and female sex.^15^ Interestingly, gut microbiota might participate in the pathogenesis of BD via epigenetic modifications of clock molecules (e.g., ARNTL).^16^ In addition to patients with current BD, gut microbiota in individuals at high risk for BD also has been investigated. No significant difference was found in gut bacterial constituents between unaffected first‐degree relatives and healthy individuals.^15^ However, in a monozygotic twin study, twins at high risk exhibited a similar pattern of gut microbial diversity to those affected by mood disorders.^17^ Taken together, these studies provided initiatory evidence that the gut microbiota may be involved in the development of BD. Nonetheless, the mood state in BD patients enrolled in these studies was not clearly classified, and the influence of psychotropic medications on the gut microbiota is not strictly controlled. Moreover, whether the gut microbial markers are helpful in disease diagnosis was not evaluated either.

The aims of this study are to characterize the gut microbiota in depressed BD patients, before and after quetiapine administration, and to study microbiota associations with clinical factors and depressive severity. Furthermore, the potential of using gut microbiota as noninvasive tool for diagnosing BD and predicting treatment outcome was also explored.

## 2 Results

### 2.1 Clinical Characteristics of the Recruited Subjects

A total of 72 BD patients and 45 healthy controls (HCs) were recruited in this study, and 20 of them with 17‐item Hamilton Depression Rating Scale (HDRS‐17) score of <14 or with missed data were excluded. Finally, 52 BD patients and 45 HCs were included for further analysis. There were no significant differences in sex and BMI between the two groups. However, HCs tended to be older (*P* < 0.05). The detailed characteristics of all individuals are shown in **Table** **1** and Table S1 (Supporting Information). Permutational multivariate analysis of variance (PERMANOVA) showed that age was weakly associated with microbial composition only in two distances (unweighted unifrac distance and spearman coefficient distance with *p* values of 0.04 and 0.002, respectively). However, the effect of BD (cohort) was much more significant than age (Table S2, Supporting Information). Among the 52 BD patients, fecal samples were re‐examined in 20 of them after quetiapine treatment. The HDRS‐17 and Montgomery‐Åsberg Depression Rating Scale (MADRS) scores were significantly decreased following quetiapine treatment in BD patients (*P* < 0.05), but no difference was observed in Young Mania Rating Scale (YMRS) scores. Demographic and clinical characteristics of the 20 treated BD patients are presented in detail in Table S3 (Supporting Information).

**Table 1:** Demographic and clinical details of recruited subjects (BD, bipolar disorder; H, healthy controls; BMI, body mass index; MADRS, Montgomery‐Åsberg Depression Rating Scale; HDRS‐17, 17‐item Hamilton Depression Rating Scale; YMRS, Young Mania Rating Scale; NOS, not otherwise specified; SD, standard deviation)

Demographic and clinical indexes		BD		H		P
		*N* = 52	**%**	*N* = 45	**%**	
Sex	Female	25	48.08	22	48.89	0.55
	Male	27	51.92	23	51.11	
Age (year, mean ± SD)		24.15 ± 9.50		36.29 ± 12.22		<0.001
BMI (kg m^−2^, mean ± SD)		21.58 ± 3.60		22.37 ± 2.91		0.77
MADRS score (mean ± SD)		28.15 ± 8.85		–		–
HDRS‐17 score (mean ± SD)		30.15 ± 8.31		–		–
YMRS score (mean ± SD)		1.87 ± 1.43		–		–
Onset age (year, mean ± SD)		19.64 ± 7.91		–		–
Duration of illness (year, mean ± SD)		4.86 ± 4.69		–		–
Bipolar diagnosis	I	12	23.08			
	II	38	73.08			
	NOS	2	3.85			
Family history	Yes	14	26.92			
	No	38	73.08			

### 2.2 Sequencing Characteristics

A total of 140 samples from all the recruited subjects were sequenced on an Illumina MiSeq sequencer. For downstream analysis, 8 222 581 qualified reads from 11 705 664 raw reads were filtered. In each sample, 20 000 reads were randomly selected. Thus, 2 788 347 reads were selected from all samples (samples with more than 10 000 reads were kept). Finally, 718 qualified Operational Taxonomy Units (OTUs) were clustered for downstream analysis. Due to their low frequencies in samples, 139 OTUs were discarded (e.g., OTU contained by no more than 2 samples or not involving more than 2 reads). A total of 96.64% of all qualified reads (not randomly selected reads) were clustered into qualified OTUs generated with randomly selected qualified reads; thus, these 718 OTUs generated by randomly selected reads could cover almost all sequences. The sequencing results from 52 untreated patients, 20 treated patients, and 45 HCs were selected for the following analysis. For details, please refer to Table S4 in the Supporting Information.

### 2.3 Gut Microbial Diversity Changes in BD Patients Compared with HCs

**Figure 1:**
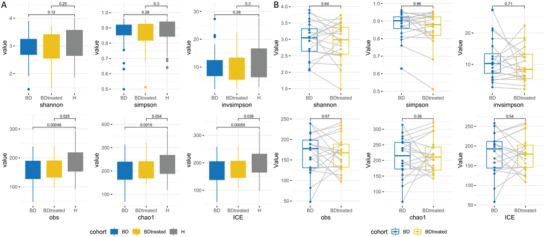
Phylogenetic diversity of the gut microbiota in untreated BD patients compared to healthy controls and in BD patients before and after treatment. A) Alpha (α) diversity in BD patients and healthy controls. Box plots depicted greater gut microbial diversity in healthy controls compared with BD patients according to the obs, chao1, and ICE indexes. The horizontal lines in the box plots represent median values; upper and lower ranges of the box represent the 75% and 25% quartiles. B) Alpha (α) diversity in untreated and treated BD patients. The x‐axis represents two cohorts; the y‐axis represents the value of each index. Each dot represents a sample. Gut microbiota diversity changes associated with quetiapine treatment were not observed according to Shannon, Simpson, invSimpson, obs, chao1, and ICE indexes.

Different diversity indexes (Shannon, Simpson, inverse Simpson [invSimpson], Obs, Chao 1 and Incidence‐based Coverage Estimators [ICE]) were used to assess gut microbiota diversity. In this study, gut microbial diversity, as estimated by Obs, Chao 1, and ICE index, was greater in HCs compared with untreated BD patients (*P* = 0.00048, 0.0015, and 0.00055, respectively). However, no diversity changes were associated with quetiapine treatment (**Figure** **1**A,B).

### 2.4 Composition at the Whole Microbiota Level

Principal coordinate analysis (PCoA) was performed with six different distances at the OTU level, focusing on the phylogenetic comparison of microbial communities. Significant differences were observed in the first five principal coordinates (PCs) between BD patients and HCs (PC1, PC2, PC3, PC4, PC5, Table S5 and Figure S1, Supporting Information; *P* < 0.05).

### 2.5 Changes of Gut Microbiota Taxonomic Composition in BD Patients

**Figure 2:**
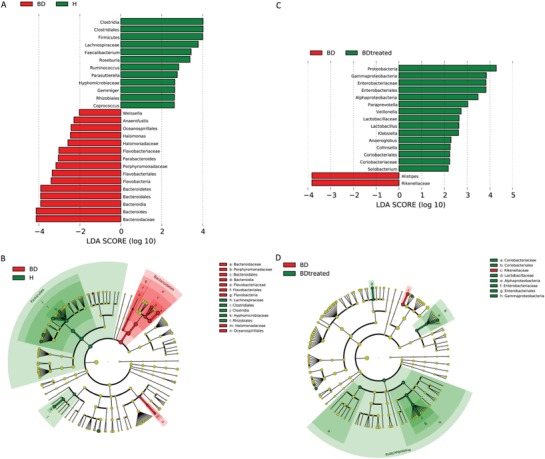
Lefse and LDA analyses revealed differences in taxonomic composition of untreated BD patients compared to healthy controls and changes in the taxonomic composition of the gut microbiota in BD patients before and after treatment with quetiapine. A) LDA scores showing significant bacterial differences between untreated BD patients (red) and healthy controls (green). B) A cladogram of different taxonomic compositions in untreated BD patients (red) and healthy controls (green). C) LDA scores showing significant bacterial differences between untreated patients (red) and treated BD patients (green). D) A cladogram of different taxonomic compositions in untreated patients (red) and treated BD patients (green).

96.7% and 88.14% of all reads were assigned into families and genera, respectively (Figure S2A, Supporting Information). At the phylum level, *Bacteroidetes*, *Firmicutes*, *Proteobacteria*, and *Actinobacteria* were the most abundant entities in the gut microbiota (Figure S2B, Supporting Information). In addition, the *Bacteroides*, *Prevotella*, *Faecalibacterium*, *Roseburia*, and *Lachnospiraceae incertae sedis* dominated the gut microbiota at the genus level (Figure S2C, Supporting Information). There was higher abundance of the *Firmicutes* phylum in HCs, while the *Bacteroidetes* group was enriched in untreated BD patients. *Parabacteroides*, *Bacteroides*, *Weissella*, and *Halomonas* abundance rates were higher in untreated BD patients compared with HCs, while the *Roseburia*, *Faecalibacterium*, *Ruminococcus*, *Gemmiger*, *Parasutterella*, and *Coprococcus* genera were more abundant in HCs (*P* < 0.05, linear discriminant analysis [LDA] score > 2; **Figure** **2**A,B). Further, we used permutational ANOVA test to validate the results from LDA effect size (Lefse) analysis and found that *Bacteroidetes* phylum, *Parabacteroides*, *Bacteroides*, and *Halomonas* genera were greatly enriched in patients, while *Firmicutes* phylum, *Roseburia*, *Faecalibacterium*, and *Coprococcus* genera were consistently higher in HCs. In addition, higher abundance of the *Proteobacteria* phylum was found following quetiapine administration in BD patients. At the genus level, *Klebsiella*, *Lactobacillus*, *Anaeroglobus*, *Collinsella*, *Paraprevotella*, *Solobacterium*, and *Veillonella* were enriched in treated BD patients, while *Alistipes* abundance was higher in untreated BD patients (*P* < 0.05, LDA score > 2; Figure 2C,D). Permutational ANOVA test showed higher *Klebsiella* and *Veillonella* in treated BD patients and no difference in phylum level between untreated and treated patients.

### 2.6 Differences of Gut Microbiota between BD‐I and BD‐II Subgroups Prior to Treatment

Among the 52 BD patients, there were 12 BD‐I patients, 38 BD‐II patients, and 2 patients classified as other types. Due to the small number of patients with other types, PCoA was performed only between BD‐I and BD‐II patients. PCoA analysis indicated that there existed no difference on the phylogenetic comparison of microbial communities between two groups (Figure S3A, Supporting Information, *P* > 0.05). However, differences of gut microbiota taxonomic compositions were observed between two subgroups. The abundance of class *Erysipelotrichia*, order *Lactobacillales*, *Erysipelotrichales*, family *Streptococcaceae*, *Erysipelotrichaceae*, genus *Streptococcus*, *Bacilli*, *Veillonella* was higher in BD‐I patients, while genus *Ruminococcus* was more abundant in BD‐II patients (*P* < 0.05, LDA score > 2; Figure S3B,C, Supporting Information). Following ANOVA testing, genus *Ruminococcus* was also enriched in BD‐II patients.

### 2.7 Function Changes Associated with Quetiapine Treatment

**Figure 3:**
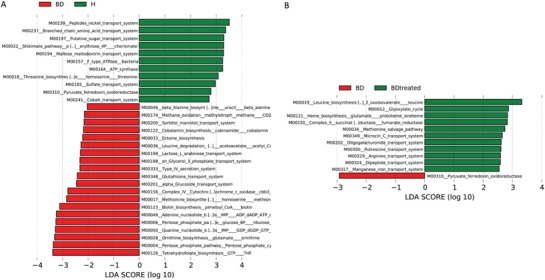
Differences in gene function in untreated BD patients compared with healthy controls, and in the BD cohort before and after treatment with quetiapine, using PICRUSt. A) Bar plots of KEGG modules are significantly different between BD patients and healthy controls. B) Bar plots of KEGG modules are significantly different in BD cohort before and after treatment with quetiapine. PICRUSt: Phylogenetic Investigation of Communities by Reconstruction of Unobserved States. KEGG: Kyoto Encyclopedia of Genes and Genomes.

Of all OTUs, 91.94% were aligned into the Phylogenetic Investigation of Communities by Reconstruction of Unobserved States (PICRUSt) built‐in reference database. Thus, a total of 5329 taxonomy and Kyoto Encyclopedia of Genes and Genomes (KEGG) orthodoxies were parsed and mapped into 118 KEGG modules. A total of 31 modules were significantly different between HCs and untreated BD patients (*P* < 0.05, LDA score > 2; **Figure** **3**A). A total of 12 modules were associated with quetiapine treatment, of which 11 and 1 were enriched in treated BD patients and untreated BD patients, respectively (Figure 3B).

### 2.8 Tree‐Based Classification Models

**Figure 4:**
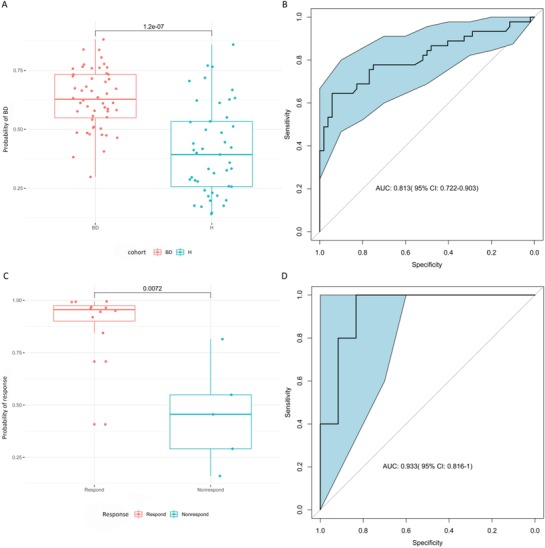
Prediction of BD based on tree‐based classification models. A,B) A significant difference was observed in BD patients and healthy controls (*P* < 0.01). A) Y‐axis represents the probability of samples predicted as “BD.” X‐axis represents ordered samples. Each dot represents a sample (red is BD, blue is H). B) Receiver operating characteristic (ROC) curves of probabilities of BD predicted by using random forest models. C,D) A significant difference was observed in responder and nonresponder BD patients (*P* < 0.01). C) Y‐axis represents the probabilities of samples predicted as “response.” X‐axis shows ordered samples. Each dot represents a sample (red is respond, blue is nonrespond). D) ROC curves of probabilities of response predicted by random forest models.

Random forest classification models were constructed with Leave‐one‐out (LOO) cross validation, using 30 genera in 52 patients and 45 controls (*P* < 0.01, Table S6, Supporting Information). The ratio of randomly generated decision trees fitting the input sample's label “BD” was termed probability of BD (POBD). The POBD value was significantly increased in the BD samples versus healthy samples (*P* = 1.2 × 10^−7^, **Figure** **4**A). A classification effect of POBD was assessed using a receiver operating characteristic (ROC) curve; the area under the ROC curve (AUC) was 0.81 (95% CI: 0.722–0.903) (Figure 4B).

We also used classification models to predict the effects of quetiapine treatment in BD patients. According to HDRS‐17 score reduction from baseline of at least 50% after a 4‐week quetiapine treatment, BD patients were divided into two subgroups, including responders and nonresponders. Because the HDRS‐17 scores for three patients were missed, 17 treated BD samples were finally included (Table S7, Supporting Information). A total of 10 genera (*P* < 0.1, Table S8, Supporting Information) in 17 samples (12 responders and 5 nonresponders) were used to construct random forest models with LOO cross validation. A significant difference was observed in responder and nonresponder BD patients (*P* < 0.01); the ROC showed an AUC of 0.93 (95% CI: 0.816–1.000; Figure 4C,D).

### 2.9 Associations of Gut Microbiota with Clinical Parameters

**Figure 5:**
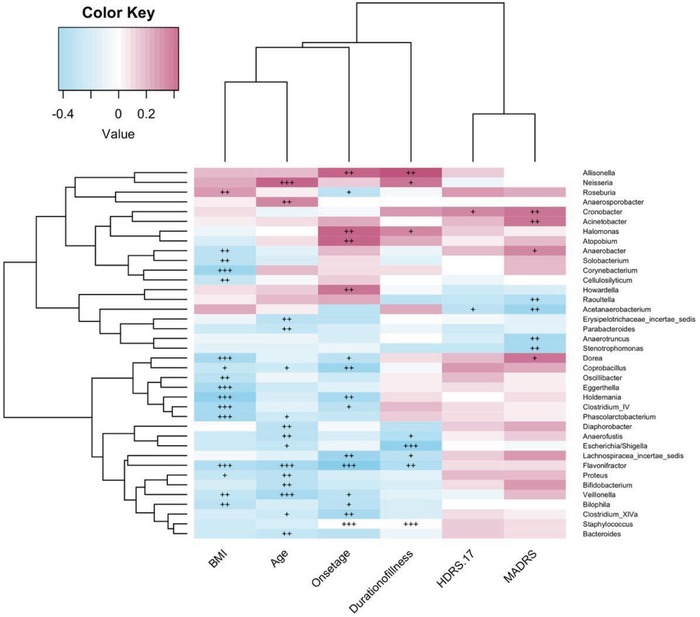
Associations of gut microbiota with clinical parameters. The heat map of Spearman's rank correlation coefficients between the gut microbiota and clinical parameters (*p* < 0.05). +, *p* < 0.10; ++, *p* < 0.05; +++, *p* < 0.01.

We also assessed the associations of clinical parameters with the gut microbiota. Regardless of quetiapine treatment, we found that the clinical parameters and severity of depression were closely associated with the abundance rates of bacterial genera in BD patients (**Figure** **5**). BMI was positively with *Roseburia* abundance but negatively correlated with *Clostridium* IV, *Dorea*, *Holdemania*, *Veillonella*, *Phascolarctobacterium*, *Eggerthella*, *Oscillibacter*, *Bilophila*, *Flavonifractor*, *Solobacterium*, and *Corynebacterium* amounts (*P* < 0.05). The duration of illness was positively correlated with *Allisonella* abundance, while negatively correlated with *Escherichia/Shigella*, *Flavonifractor*, *Staphylococcus* abundance (*P* < 0.05). In addition, MADRS scores were negatively correlated with the levels of *Acetanaerobacterium*, *Stenotrophomonas*, *Anaerotruncus* and *Raoultella*,but positively correlated with *Acinetobacter* and *Cronobacter* (*P* < 0.05).

## 3 Discussion

This study characterized the gut microbiota in depressed BD patients compared with HCs, and before and after quetiapine treatment. Moreover, associations of gut microbiota with clinical parameters and the severity of depression were investigated. The results demonstrated that the gut microbiota's taxonomic composition and diversity were significantly different in BD patients. Gut microbiota compositions in untreated BD patients and HCs were dominantly characterized by *Bacteroidetes* and *Firmicutes*, respectively. Moreover, we found that several butyrate‐producing genera were abundant in HCs compared to untreated BD patients. This study also revealed that the amounts of specific genera were correlated with clinical parameters and depressive severity. Notably, our results indicated that BD patients and HCs could potentially be distinguished by the gut microbiota, and treatment outcome might also be predicted by microbial markers.

As shown in the results, greater diversity of gut microbiota was found in HCs compared with BD patients; however, quetiapine treatment did not change the diversity in our samples. Gut microbiota diversity could be affected by various factors, such as diet, health status, and age, the richness in bacterial diversity is possibly protective from metabolic and autoimmune diseases.^18^ Although some studies reported no significant difference in gut microbiota diversity between major depressive disorder (MDD) patients and healthy individuals,^19^ while others found it was greater in MDD patients.^20^ A previous study has assessed the interaction between atypical antipsychotic (AAP) and gut microbiota in BD patients, and showed gender‐specific reduction of species richness in AAP‐treated females, which may be associated with metabolic factors.^14^

Our results demonstrated that the microbial composition in depressed BD patients was clearly different from HCs. Phylum *Firmicutes* were more abundant in healthy individuals, while greater amounts of *Bacteroidetes* were found in untreated depressed BD patients, in accordance with a previous study in MDD patients.^20^ Of note, compared to HCs, decreased abundance of various butyrate‐producing bacteria was found in untreated BD patients, including genera *Roseburia*, *Faecalibacterium*, and *Coprococcus*. These bacteria can produce short‐chain fatty acids, such as butyrate, acetic acid, and valeric acid.^21^, ^22^ Butyrate, one of the main products of colonized microbiota, serves as a major energy source for colonocytes,^23^ and plays an important role in immune accommodation, gut barrier regulation, gut metabolism, and energy modulation.^22^, ^24^ Butyrate has been reported to be beneficial for many systemic disorders, such as hemoglobinopathy, genetic metabolic diseases, hypercholesterolemia, insulin resistance, and ischemic stroke.^23^ Furthermore, butyrate in the central nervous system can affect the function of hippocampus and promote the expression of BDNF, which has been shown to have antidepressant‐like effects in animal models.^25^ Therefore, inadequate butyrate‐producing bacteria in BD patients may contribute to the disease pathology. Following quetiapine treatment, gut microbial composition was remarkably changed. *Klebsiella* and *Veillonella* genera were abundant in treated patients. Previous studies observed higher levels of *Klebsiella* and *Veillonella* in MDD^26^ and BD^27^ patients, respectively. These findings provide primary evidence that the gut ecosystem in BD patients is different from health individuals, even if the depressive symptoms are alleviated. Some possible explanations for the alterations of gut microbiota in treated BD patients need to be mentioned. On the one hand, patients with spontaneous remission and drug‐assisted remission may have different gut microbial communities. On the other hand, quetiapine treatment increases the risk of metabolic disturbance in susceptible patients, which may be associated with specific bacteria in the gut.

We further investigated the association between relevant clinical parameters and depressive severity with gut microbiota. Among BD patients, BMI was positively associated with *Roseburia* abundance, but negatively correlated with *Clostridium* IV, *Dorea*, *Holdemania*, *Veillonella*, *Phascolarctobacterium*, *Eggerthella*, *Oscillibacter*, *Bilophila*, *Flavonifractor*, *Solobacterium*, and *Corynebacterium*. In patients with anorexia nervosa, *Clostridium* abundance was shown to be negatively correlated with psychopathological scores.^28^ An animal study showed that increased abundance in *Clostridium* was related to antidepressant‐like effects in susceptible mice.^29^ Furthermore, *Clostridium* IV abundance was increased after weight loss.^30^ These findings indicated that *Clostridium* IV might be involved in the development of BD through metabolic pathways. In addition, *Phascolarctobacterium* genus was found to be correlated with positive mood in healthy adults.^31^ *Holdemania* genus is associated with glucose metabolism and metabolic syndrome,^32^ which is highly prevalent in BD patients.^33^ Therefore, gut microbiota may participate in the metabolic perturbations in BD individuals.

Previous studies have reported a negative association between *Faecalibacterium* abundance and disease severity in MDD and BD patients.^12^, ^20^ In our study, MADRS scores were negatively correlated with *Acetanaerobacterium*, *Stenotrophomonas*, *Anaerotruncus*, and *Raoultella* levels, and positively correlated with *Acinetobacter* and *Cronobacter* levels. *Acetanaerobacterium* and *Anaerotruncus* all belong to *Ruminococcaceae* family, which was decreased in BD patients compared with HCs.^13^ We also found higher abundance of *Ruminococcaceae* family in HCs. *Ruminococcaceae* family was reported to be correlated with energy metabolism pathways, including gluconeogenesis, glycolysis, and the pentose phosphate pathways.^34^ Therefore, lower *Ruminococcaceae* in BD patients was possibly related to abnormal glucose metabolism. Genera *Stenotrophomonas* and *Raoultella* were both members of *Gammaproteobacteria* class, and treated patients were riched in *Gammaproteobacteria* compared with untreated patients in our study. Hence, the bacteria clades associated with depressive severity may link to energy metabolism and pharmacotherapy.

Notably, this study is the first to show that BD patients and HCs can be possibly distinguished by gut microbiota. Our study has achieved satisfied classification efficacy for distinguishing BD patients from HCs based on random forest classification models. Previous studies had shown that gut microbiota could be regarded as potential noninvasive biomarkers for diagnosing nonpsychiatric diseases, such as hepatocellular carcinoma,^35^ type 2 diabetes,^36^ and colorectal cancer.^37^ Our study used optimal 30 OTUs markers based on 16S rRNA sequences, and achieved a high accuracy (AUC = 0.81) between BD patients and HCs, indicating a powerful classification efficacy. Furthermore, the predicted effects of quetiapine treatment in BD patients were also assessed and achieved an AUC of 0.93 between responders and nonresponders. These findings provide important evidence that gut microbiota‐targeted biomarkers were potentially helpful in predicting the treatment outcome of BD. Nevertheless, the microbial marker‐based models still need to be validated in larger samples.

Several limitations of this study should be mentioned. Gut microbiota can be influenced by multiple variables. Although use of antibiotics, probiotics, and prebiotics was not allowed in our study, participants did not receive a standardized diet. Regional variation could also be an influential factor of gut microbiota.^38^ However, the regional sources of our samples were not controlled. Of note, the age of participants was not matched between patients and controls. Although we found a weak association of age with gut microbial composition, the effect of cohort was comparatively more significant. Another limitation was the follow‐up rate in our patients, resulting in re‐examining only a subset of patients. Although this study evaluated the impact of quetiapine monotherapy on the gut ecosystem, patients were only assessed at two time points. Therefore, our study cannot explain whether the changes in microbiota are trait‐ or state‐related feature. Longitudinal design with patients in mania or remission is needed to clarify the cause–effect relationship between BD and gut microbiota.

## 4 Conclusions

This study indicated significant alterations in gut microbiota in depressed drug‐free BD patients, which may be related to multiple factors, including metabolic pathways, depressive severity, and pharmacological treatment. Short‐term quetiapine treatment failed to draw the microbial ecosystem of BD patients close to that of healthy individuals. Notably, gut microbial markers might be helpful for classifying BD and predicting treatment outcome. These findings provide further evidence that the microbiota–gut–brain axis is involved in BD pathogenesis. Future investigations should dig deeper to clarify the connections between gut microbiota and brain function in BD patients.

## 5 Experimental Section

*Participants*: This study was approved by the Institutional Review Board of the First Affiliated Hospital, School of Medicine of Zhejiang University (reference number #2017‐397), and performed in accordance with the Helsinki Declaration. Written informed consent was obtained from all participants. BD patients with a current depressive episode were recruited from the Psychiatric Department of the First Affiliated Hospital, School of Medicine, Zhejiang University. Diagnoses according to DSM‐IV‐TR criteria for BD‐I, BD‐II and BD not otherwise specified (NOS) were confirmed by structured psychiatric interview, using the Mini International Neuropsychiatric Interview (M.I.N.I.).^39^ MADRS^40^ and HDRS‐17^41^ were used to evaluate the severity of depression, and YMRS^42^ was used to assess the severity of mania. The HDRS‐17 score ≥14 was regarded as moderate to severe severity of depression, and was set as the threshold for inclusion. All patients were first episode or psychotropic drug free for at least 3 months. Patients with any other psychiatric comorbidities were excluded. HCs with no psychiatric disorder or a family history of psychiatric disorder were recruited from local communities. For BD and HC subjects, other exclusion criteria were severe physical diseases involving the heart, lung, liver, and gut, as well as acute and chronic infections and substance abuse disorders. Moreover, pregnant or breast‐feeding female subjects were excluded. Use of antibiotics, probiotics, or prebiotics for less than 4 weeks before sample collection was not allowed. At baseline, fecal samples were collected from all subjects. All patients received 4 weeks of quetiapine treatment (maintenance dose, 200–300 mg d^−1^), following which fecal samples were re‐examined follow‐up patients.

*Clinical Characteristics Analysis*: Analysis of demographic and clinical data was conducted with the SPSS 20.0 statistical package (IBM, IL, USA), using two‐tailed student's t‐test or chi‐square test, as appropriate. PERMANOVA was used to test association between microbial community and clinical characteristics. *P* < 0.05 was set as statistically significant.

*Fecal Sample Collection and DNA Extraction*: Fecal samples from all subjects were stored at −80 °C within 0.5 hour after collection in the State Key Laboratory for Diagnosis and Treatment of Infectious Diseases, the First Affiliated Hospital, School of Medicine, Zhejiang University. DNA was extracted using the PSP Spin Stool DNA Plus Kit (Stratec, Berlin, Germany) according to the manufacturer's instructions. Isolated DNA was quantified using a Qubit 2.0 Fluorometer (Invitrogen, Carlsbad, CA, USA). DNA integrity was assessed by agarose gel electrophoresis and DNA was stored at −20 °C before microbial MiSeq sequencing.

*PCR and Sequencing*: The V3–V4 region of 16S rRNA was amplified by PCR with primers 341F 5′‐barcode‐CCTACGGGNGGCWGCAG‐3′ and 785R 5′‐GACTACHVGGGTATCTAATCC‐3′, as previously described.^43^ High‐throughput sequencing was performed on an Illumina MiSeq instrument according to the manufacturer's instructions. Raw Illumina read data have been deposited in the European Nucleotide Archive database (Study accession Number: PRJEB23500).

*Sequence Processing*: All pair‐end sequences were merged with Flash v1.2.11^44^ using default parameters except “‐m 10 ‐M 300 × 0.15 ‐O”; then, custom perl scripts were used to filter and assign overlapped reads into different samples: 1) no mismatch in primers and barcode regions (compared with barcode‐primer sequences in PCR experiments) allowed; 2) a maximum mismatch rate in the overlapping region no more than 0.05; 3) maximum mismatch bases in the overlapping region no more than 5; 4) no ambiguous bases allowed in all reads; 5) minimum and maximum lengths of read (without barcodes and primers) of 100 bp and 550 bp, respectively. Next, chimeras were removed by using Uchime^45^ with the “‐strand plus” parameter and the recommended built‐in sequence (http://drive5.com/uchime/gold.fa) as reference. Filtered reads were used for downstream analysis.

*OTU and KEGG Function Module Profiles*: Randomly selected sequences from all samples were clustered into OTUs using the "Usearch ‐cluster_otus” function^46^ with default parameters (unique reads with abundance no more than two were filtered out). Then, OTU profiles were constructed by aligning randomly selected sequences with representative OTU sequences as reference using the “Usearch ‐usearch_global” function with 97% cutoff (‐id 0.97). All OTU sequences were annotated using RDP classier version 2.12^47^ with a significance level of 0.80 (‐c 0.8 ‐f fixrank); sequences which could not be assigned into a specific taxonomy level were labeled as “unclassified.” PICRUSt version 1.0.0^48^ was used to generate KEGG ontology profile. Human version 0.99^49^ was used to assign ontology into the KEGG pathway at the module level.

*Alpha Diversity and Distance Calculation*: Relative abundance in OTU profiles was used to determine alpha diversity and distance matrix for all samples. The Vegan version 2.4.4^50^ package was used to determine Shannon, Simpson, and invSimpson indexes. The Simpson's index in this study was a variant of the original Simpson's diversity index (D), and obtained as 1‐D. The number of OTUs in each sample was calculated as Obs index. The Fossil^51^ version 0.3.7 package was used to determine Chao 1 and ICE index. A custom R script (http://enterotyping.embl.de/enterotypes.html) was used to determine JSD. Spearman coefficient distance was calculated in the R program as follows: 1‐ dist(cor(dat),method =*“spearman”*), where dat is the relative abundance of the OTU profile. MUSCLE v3.8.31^52^ was used to perform multiple alignments of representative OTU sequences, with FastTree version 2.1.8^53^ employed to generate a phylogenetic tree with the generalized time‐reversible model. The phyloseq package version 1.20.0^54^ was used to obtain the UniFrac distance (weighted and unweighted), with relative OTU abundance and rooted phylogenetic tree. To explore whether differences of gut microbiota exist between different types of BD, we use PCoA to calculate the beta diversity by weighted UniFrac, unweighted UniFrac, hellinger and JSD.

*Statistical Analysis*: Wilcoxon rank sum test was used to perform hypothesis test of alpha diversity and PCs among different cohorts. Lefse version 1.0^55^ was used to identify taxa and KEGG modules, which may be significantly associated with quetiapine treatment or cohorts (LDA score (log10) = 2 as cutoff value). For paired samples (two samples before and after quetiapine treatment, from the same patients), *p*‐values by paired Wilcoxon rank sum test were used, instead of Lefse build‐in hypothesis test. Permutational ANOVA test^56^ was employed to determine the significance of difference between health and BD patient groups (i.e., HC vs BD; BD pretreatment vs BD posttreatment; BD‐I vs BD‐II). Statistical significance of each test was determined by comparing the actual F test result from ANOVA to that obtained from 10 000 random permutations of the individuals between the compared groups of each test. All these analyzes were conducted with R package.^57^ Spearman's rho statistic was used to estimate the association between clinical parameters and taxons.

*Random Forest Classification Models*: Random Forest 4.6‐12^58^ was used to build classification models using profiles of genera with significant differences between the two cohorts. The term “POBD” was used as samples with probabilities could be classified as “BD.” The LOO cross validation mode was used; thus, in the training stage, one in all *m* samples would be trained in *m* training models. Finally, mean POBD was used as the final training POBD. The evaluation of the random forest classification models was performed by ROC curve (R 3.3.0, pROC package), and AUC was used to assess the ROC effect. The same method was used to predict the treatment outcome in BD patients.

## Conflict of Interest

The authors declare no conflict of interest.

## Supporting information

Supplementary
