# Supplementary material for: Gut Microbiota Changes in Patients with Bipolar Depression
Source: Adv Sci (Weinh). 2019 May 15;6(14):1900752. doi: 10.1002/advs.201900752 (PMC6662053; doi:10.1002/advs.201900752)
Supplement: Supplementary file 1 — Supplementary [file ADVS-6-1900752-s001.pdf]

Copyright WILEY-VCH Verlag GmbH & Co. KGaA, 69469 Weinheim, Germany, 2019.

# Supporting Information

for *Adv. Sci.,* DOI: 10.1002/advs.201900752

## Gut Microbiota Changes in Patients with Bipolar Depression

*Shaohua Hu, Ang Li, Tingting Huang, Jianbo Lai, Jingjing Li, M. Elizabeth Sublette, Haifeng Lu, Qiaoqiao Lu, Yanli Du, Zhiying Hu, Chee H. Ng, Hua Zhang, Jing Lu, Tingting Mou, Shaojia Lu, Dandan Wang, Jinfeng Duan, Jianbo Hu, Manli Huang, Ning Wei, Weihua Zhou, Liemin Ruan, Ming D. Li,* and Yi Xu**

## SUPPLEMENTARY FIGURES

**Figure S1: Principle coordinates of microbial communities by principal coordinate analysis (PCoA) among three cohorts.** (A-F) PCoA of unweighted UniFrac, weighted UniFrac, Hellinger, JSD, Pearson and Spearman distances at the OTU level showing significant differences in the first three principal coordinates between BD patients and healthy controls.

**Figure S2: Taxonomy resolution of gut microbiota among healthy controls, untreated and treated BD patients.** (A) Taxonomy resolution in all samples; each bar represents ratio of reads annotated at that level. (B) Relative abundance levels of the phyla present in samples from healthy controls, untreated and treated BD patients. *Bacteroidetes*, *Firmicutes*, *Proteobacteria*, and *Actinobacteria* were the most abundant in the gut microbiota. (C) Relative abundance levels of genera present in samples from healthy controls, untreated and treated BD patients. The *Bacteroides*, *Prevotella*, *Faecalibacterium*, *Roseburia* and *Lachnospiracea incertae sedis* genera dominate the gut microbiota.

**Figure S3: Differences of gut microbiota between BD-I and BD-II subgroups.**

(A) Principle coordinates of microbial communities by principal coordinate analysis (PCoA) between BD-I and BD-II patients, indicating no difference between BD-I and BD-II patients. (B) LDA scores showed significant bacterial differences between BD-I (red) and BD-II (green) subgroups. (C) A cladogram of different taxonomic compositions between BD-I (red) and BD-II (green) subgroups.

## SUPPLEMENTARY TABLES

**Table S1. Detailed characteristics of the recruited subjects**

**Table S2. PERMANOVA results by using distances and characteristics of recruited individuals**

**Table S3. Demographic and clinical details of BD patients following quetiapine treatment**

**Table S4. 16S rRNA Sequencing Data from the Recruited Subjects**

**Table S5. Wicoxon rank sum test of principle coordinates in BD patients and healthy controls**

**Table S6. Genera to construct random forest models to distinguish BD patients and healthy controls**

**Table S7. HDRS-17 and MADRS score changes following quetiapine treatment in BD patients**

**Table S8. Genera to construct random forest models to distinguish quetiapine treatment response in BD patients**

**Figure S1: Principle coordinates of microbial communities by principal coordinate analysis (PCoA) among three cohorts.**

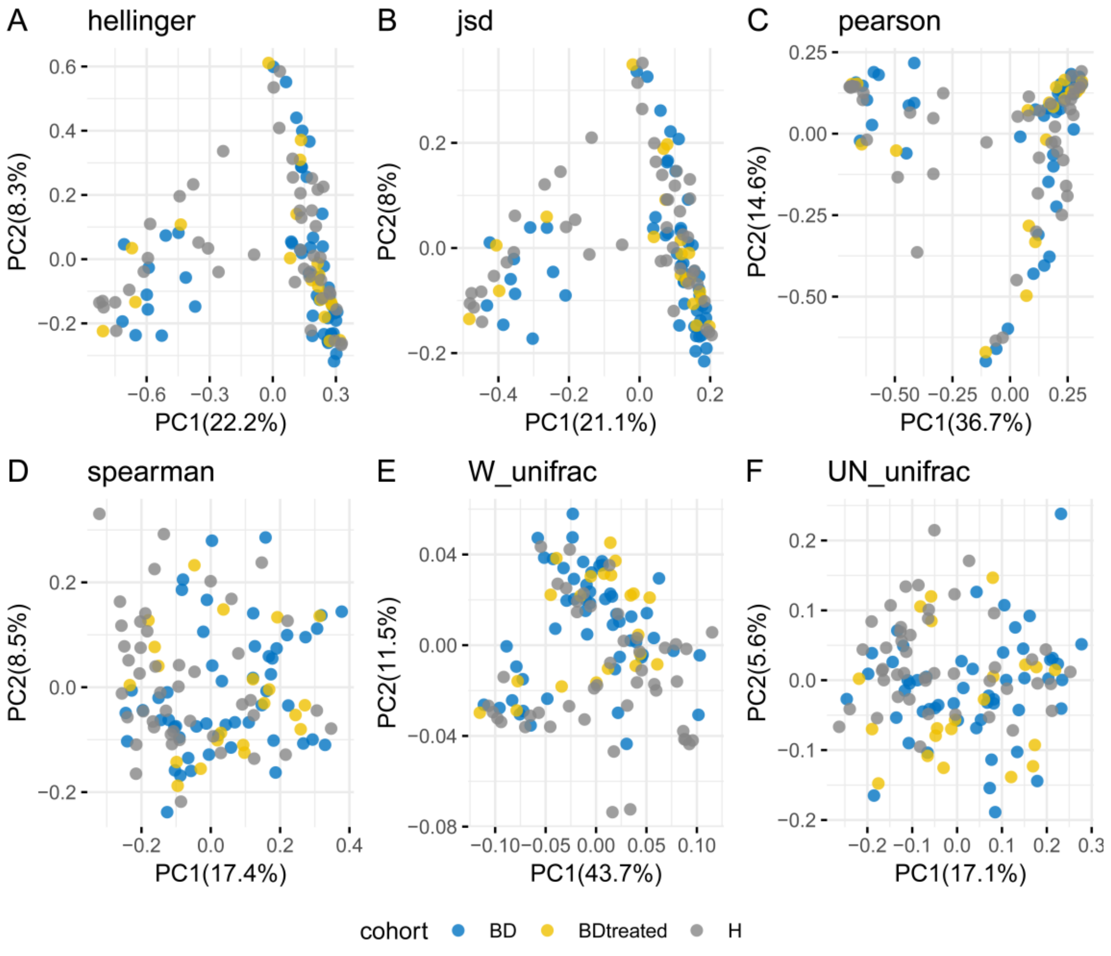

**Figure S2: Taxonomy resolution of gut microbiota among healthy controls, untreated and treated BD patients.**

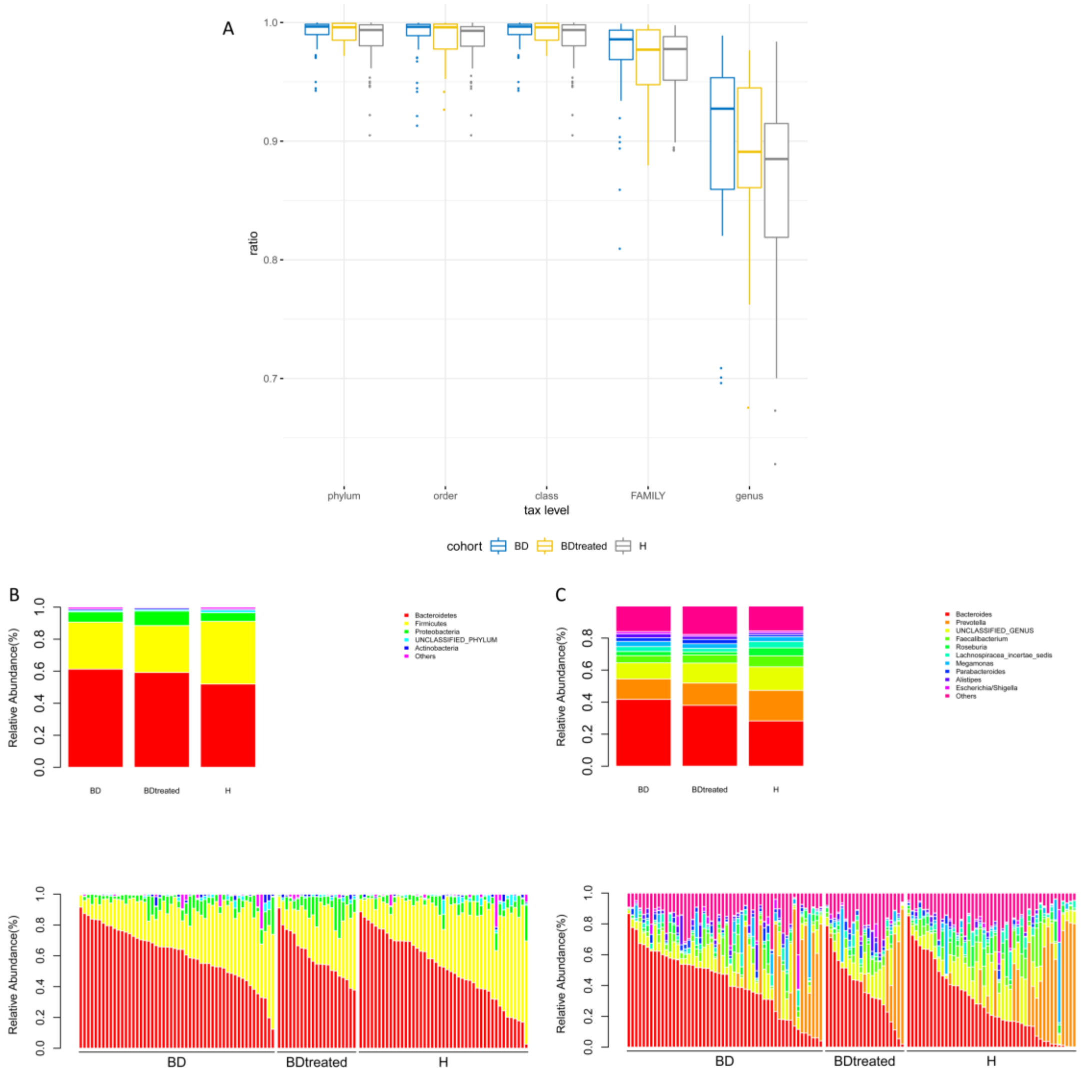

**Figure S3: Differences of gut microbiota between BD-I and BD-II subgroups.**

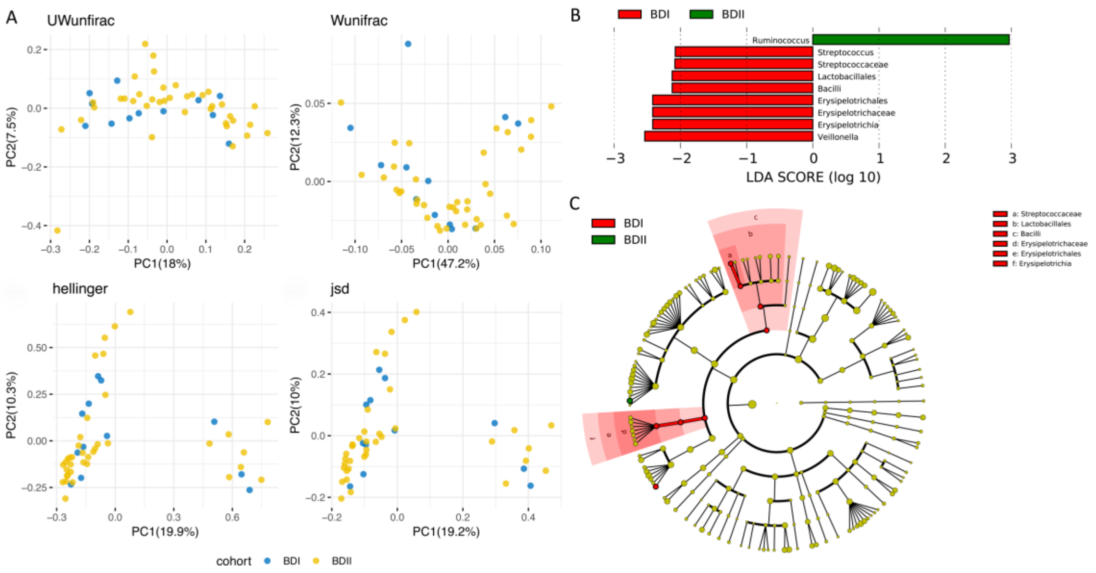


| Table S1. Detailed characteristics of the recruited subjects | | | | | | | | | | | | |
|---|---|---|---|---|---|---|---|---|---|---|---|---|
| **Number** | **Group** | **Gender** | **Age (year)** | **BMI** | **Family history** | **Onset age (year)** | **Duration of illness** | **Bipolar diagnosis** | **MADRS** | **HDRS-17** | **YMRS** | |
| 1 | BD | Female | 40 | 25.59 | Y | 21 | 19 | I | 35 | 39 | 0 | |
| 2 | BD | Male | 40 | 21.47 | N | 34 | 6 | II | 25 | 27 | 3 | |
| 3 | BD | Male | 41 | 23.88 | N | 41 | 0.5 | II | 23 | 29 | 2 | |
| 4 | BD | Male | 16 | 17.72 | N | 14 | 1.5 | II | 37 | 36 | 2 | |

| | | | | | | | | | | | | |
|---|---|---|---|---|---|---|---|---|---|---|---|---|
| 5 | BD | Female | 14 | 26.37 | N | 12 | 2 | I | 32 | 34 | 2 | |
| 6 | BD | Female | NA | NA | NA | NA | NA | NA | NA | NA | NA | Discard |
| 7 | BD | Male | 22 | 23.15 | N | 16 | 6 | II | 25 | 43 | 1 | |
| 8 | BD | Male | 16 | 28.73 | Y | 15 | 1 | II | 26 | 24 | 2 | |
| 9 | BD | Male | 34 | 22.91 | Y | 33 | 1 | II | 23 | 31 | 3 | |
| 10 | BD | Female | 19 | 22.84 | N | 12 | 7 | NA | 14 | 15 | 1 | |
| 11 | BD | Female | 26 | 17.58 | N | 24 | 2 | I | 33 | 31 | 4 | |
| 12 | BD | Male | 15 | 17.92 | N | 15 | 0.75 | I | 32 | 37 | 4 | |
| 13 | BD | Male | NA | NA | NA | NA | NA | NA | NA | NA | NA | Discard |
| 14 | BD | Male | 27 | 28.08 | N | 18 | 9 | II | 25 | 33 | 3 | |
| 15 | BD | Female | 58 | 22.58 | N | 48 | 10 | I | 46 | 50 | 0 | |
| 16 | BD | Male | 28 | NA | N | NA | NA | I | 23 | 30 | 1 | |
| 17 | BD | Female | NA | NA | NA | NA | NA | NA | NA | NA | NA | Discard |
| 18 | BD | Female | 21 | 16.82 | N | 16 | 5 | I | 21 | 24 | 3 | |
| 19 | BD | Female | 43 | 25.81 | N | 26 | 17 | II | 22 | 26 | 3 | |
| 20 | BD | Male | 23 | 22.49 | N | 16 | 7 | II | 25 | 28 | 3 | |
| 21 | BD | Male | 15 | 16.71 | N | NA | NA | II | 51 | 39 | 2 | |
| 22 | BD | Male | 16 | 16.36 | N | 12 | 4 | II | 30 | 40 | 1 | |
| 23 | BD | Female | 38 | 21.37 | Y | 23 | 15 | I | 20 | 18 | 1 | |
| 24 | BD | Male | 18 | 24.21 | N | 12 | 6 | I | 26 | 20 | 3 | |
| 25 | BD | Female | 17 | 20.58 | N | 14 | 3 | II | 35 | 25 | 4 | |
| 26 | BD | Male | 16 | 22.23 | N | 14 | 1.5 | II | 17 | 22 | 1 | |

| | | | | | | | | | | | | |
|---|---|---|---|---|---|---|---|---|---|---|---|---|
| 27 | BD | Male | 16 | 30.45 | N | 16 | 0.25 | NA | 27 | 30 | 4 | |
| 28 | BD | Male | 22 | 19.53 | N | 19 | 3 | II | 41 | 35 | 3 | |
| 29 | BD | Male | NA | NA | NA | NA | NA | NA | NA | NA | NA | Discard |
| 30 | BD | Male | 19 | 22.1 | Y | 16 | 3 | II | 15 | 15 | 2 | |
| 31 | BD | Female | 40 | 20.31 | Y | 29 | 11 | II | 28 | 33 | 2 | |
| 32 | BD | Male | 19 | 19 | N | 17 | 2 | II | 17 | 13 | 0 | Discard |
| 33 | BD | Female | 16 | 24.22 | Y | 14 | 2 | II | 16 | 18 | 2 | |
| 34 | BD | Female | NA | NA | NA | NA | NA | NA | NA | NA | NA | Discard |
| 35 | BD | Female | 25 | 16.8 | N | 23 | 2 | II | 32 | 34 | 2 | |
| 36 | BD | Female | 18 | 18.44 | N | 15 | 3 | II | 30 | 29 | 0 | |
| 37 | BD | Female | 17 | 16.41 | N | 15 | 2 | II | 27 | 24 | 0 | |
| 38 | BD | Male | 17 | 21.22 | Y | 15 | 2 | II | 38 | 40 | 0 | |
| 39 | BD | Male | 30 | 23.84 | N | 16 | 14 | I | 22 | 22 | 2 | |
| 40 | BD | Male | 27 | 23.3 | N | 16 | 11 | II | 26 | 38 | 5 | |
| 41 | BD | Male | NA | NA | NA | NA | NA | NA | NA | NA | NA | Discard |
| 42 | BD | Female | NA | NA | NA | NA | NA | NA | NA | NA | NA | Discard |
| 43 | BD | Male | 17 | 19.75 | N | 15 | 2 | II | 11 | 8 | 4 | Discard |
| 44 | BD | Female | 24 | 21.37 | Y | 16 | 8 | II | 44 | 39 | 1 | |
| 45 | BD | Female | NA | NA | NA | NA | NA | NA | NA | NA | NA | Discard |
| 46 | BD | Male | NA | NA | NA | NA | NA | NA | NA | NA | NA | Discard |
| 47 | BD | Female | 20 | 19.78 | N | 19 | 1 | II | 7 | 5 | 0 | Discard |
| 48 | BD | Male | 34 | 23.67 | Y | 7 | 30 | I | 4 | 7 | 0 | Discard |

| | | | | | | | | | | | | |
|---|---|---|---|---|---|---|---|---|---|---|---|---|
| 49 | BD | Female | 30 | 21.5 | N | 29 | 1 | II | 15 | 19 | 0 | |
| 50 | BD | Female | NA | NA | NA | NA | NA | NA | NA | NA | NA | Discard |
| 51 | BD | Male | 31 | 27.04 | Y | 21 | 10 | I | 28 | 35 | 4 | |
| 52 | BD | Female | NA | NA | NA | NA | NA | NA | NA | NA | NA | Discard |
| 53 | BD | Female | 22 | 17.96 | N | 21 | 1 | II | 28 | 30 | 3 | |
| 54 | BD | Male | 16 | 17.3 | N | NA | NA | II | 30 | 36 | 0 | |
| 55 | BD | Male | 21 | 18.43 | N | 15 | 3 | II | 32 | 35 | 0 | |
| 56 | BD | Female | 21 | 23.44 | N | NA | NA | II | 38 | 37 | 3 | |
| 57 | BD | Female | 22 | 21.77 | N | 20 | 2 | II | 6 | 6 | 0 | Discard |
| 58 | BD | Male | 17 | 20.05 | N | 15 | 2 | II | 16 | 13 | 0 | Discard |
| 59 | BD | Female | 29 | 17.53 | N | 22 | 7 | II | 45 | 36 | 0 | |
| 60 | BD | Female | 14 | 20.43 | N | 13 | 0.25 | II | 19 | 22 | 4 | |
| 61 | BD | Male | 20 | 21.78 | N | 15 | 5 | II | 48 | 46 | 0 | |
| 62 | BD | Male | 20 | 17.43 | N | 19 | 1 | II | 20 | 24 | 3 | |
| 63 | BD | Male | 41 | 13.86 | NA | NA | NA | NA | NA | NA | NA | Discard |
| 64 | BD | Male | 21 | 18.94 | Y | 21 | 0.5 | II | NA | NA | NA | Discard |
| 65 | BD | Female | 20 | 18.2 | Y | 15 | 5 | II | 21 | 29 | 0 | |
| 66 | BD | Male | 39 | 27.73 | Y | 34 | 5 | II | 28 | 26 | 0 | |
| 67 | BD | Male | 27 | 25.86 | N | 24 | 3 | II | 16 | 12 | 3 | Discard |
| 68 | BD | Female | 26 | 25.95 | N | 25 | 1 | I | 26 | 40 | 2 | |
| 69 | BD | Male | 17 | 18.94 | Y | 17 | 0.25 | II | 17 | 18 | 3 | |
| 70 | BD | Female | 17 | 21.45 | N | 14 | 3 | II | 34 | 37 | 1 | |

| | | | | | | | | | | | | |
|---|---|---|---|---|---|---|---|---|---|---|---|---|
| 71 | BD | Female | 17 | 22.21 | N | 17 | 0.25 | II | 21 | 20 | 0 | |
| 72 | BD | Female | 22 | 20.03 | Y | NA | NA | II | 20 | 22 | 0 | |
| 73 | H | Female | 35 | 19.31 | N | / | / | / | / | / | / | |
| 74 | H | Female | 49 | 23.24 | N | / | / | / | / | / | / | |
| 75 | H | Female | 43 | NA | N | / | / | / | / | / | / | |
| 76 | H | Male | 32 | 23.94 | N | / | / | / | / | / | / | |
| 77 | H | Female | 28 | 21.99 | N | / | / | / | / | / | / | |
| 78 | H | Female | 38 | 21.26 | N | / | / | / | / | / | / | |
| 79 | H | Male | 49 | 18.86 | N | / | / | / | / | / | / | |
| 80 | H | Male | 54 | 27.28 | N | / | / | / | / | / | / | |
| 81 | H | Male | 50 | NA | N | / | / | / | / | / | / | |
| 82 | H | Female | 36 | 19.25 | N | / | / | / | / | / | / | |
| 83 | H | Male | 38 | NA | N | / | / | / | / | / | / | |
| 84 | H | Female | 38 | 19.39 | N | / | / | / | / | / | / | |
| 85 | H | Male | 31 | 21.29 | N | / | / | / | / | / | / | |
| 86 | H | Male | 59 | 26.73 | N | / | / | / | / | / | / | |
| 87 | H | Female | 61 | 22.29 | N | / | / | / | / | / | / | |
| 88 | H | Female | 28 | 18.76 | N | / | / | / | / | / | / | |
| 89 | H | Female | 22 | 20.28 | N | / | / | / | / | / | / | |
| 90 | H | Male | 24 | 24.22 | N | / | / | / | / | / | / | |
| 91 | H | Female | 24 | 17.78 | N | / | / | / | / | / | / | |
| 92 | H | Male | 34 | 20.38 | N | / | / | / | / | / | / | |
| 93 | H | Fem | 24 | 20. | N | / | / | / | / | / | / | |

| | | | | | | | | | | | | |
|---|---|---|---|---|---|---|---|---|---|---|---|---|
| | | ale | | 31 | | | | | | | | |
| 94 | H | Female | 46 | 24.65 | N | / | / | / | / | / | / | |
| 95 | H | Female | 24 | 20.81 | N | / | / | / | / | / | / | |
| 96 | H | Male | 17 | 20.42 | N | / | / | / | / | / | / | |
| 97 | H | Male | 18 | 20.28 | N | / | / | / | / | / | / | |
| 98 | H | Male | 17 | 27.68 | N | / | / | / | / | / | / | |
| 99 | H | Male | 17 | 21.79 | N | / | / | / | / | / | / | |
| 100 | H | Male | 18 | 20.16 | N | / | / | / | / | / | / | |
| 101 | H | Male | 38 | 25.35 | N | / | / | / | / | / | / | |
| 102 | H | Male | 30 | 29.41 | N | / | / | / | / | / | / | |
| 103 | H | Male | 42 | 25.69 | N | / | / | / | / | / | / | |
| 104 | H | Female | 37 | 23.05 | N | / | / | / | / | / | / | |
| 105 | H | Male | 25 | 20.76 | N | / | / | / | / | / | / | |
| 106 | H | Female | 60 | 20.03 | N | / | / | / | / | / | / | |
| 107 | H | Female | 47 | 26.04 | N | / | / | / | / | / | / | |
| 108 | H | Male | 40 | 21.63 | N | / | / | / | / | / | / | |
| 109 | H | Female | 31 | 20.31 | N | / | / | / | / | / | / | |
| 110 | H | Female | 26 | 19.48 | N | / | / | / | / | / | / | |
| 111 | H | Male | 54 | 25.16 | N | / | / | / | / | / | / | |
| 112 | H | Male | 46 | 22.73 | N | / | / | / | / | / | / | |
| 113 | H | Female | 45 | 24.6 | N | / | / | / | / | / | / | |
| 114 | H | Male | 38 | 27.66 | N | / | / | / | / | / | / | |

| | | | | | | | | | | | | |
|---|---|---|---|---|---|---|---|---|---|---|---|---|
| 115 | H | Male | 34 | 22.75 | N | / | / | / | / | / | / | |
| 116 | H | Female | 49 | 20.13 | N | / | / | / | / | / | / | |
| 117 | H | Female | 37 | NA | N | / | / | / | / | / | / | |

**Table S2. PERMANOVA results by using distances and characteristics of recruited individuals**

| | Cohort (P) | Age (P) | BMI (P) | Gender (P) | Residual ($R^2$) |
|---|---|---|---|---|---|
| **Weighted Unifrac** | 0.0252 | 0.2349 | 0.1615 | 0.0024 | 0.88386 |
| **Unweighted Unifrac** | 0.0003 | 0.04015 | 0.1947 | 0.1054 | 0.92159 |
| **Spearman** | 1e-4 | 0.0025 | 0.1107 | 0.0233 | 0.88351 |
| **Hellinger** | 0.011899 | 0.078592 | 0.023598 | 0.006 | 0.91146 |
| **JSD** | 0.012 | 0.088291 | 0.02 | 0.006 | 0.91465 |
| **Pearson** | 0.059194 | 0.1225 | 0.011 | 0.006 | 0.87986 |

| Table S3. Demographic and clinical details of BD patients following quatiapine treatment | | | | |
|---|---|---|---|---|
| Demographic and Clinical Indexes | | BD patients following quatiapine treatment | | |
| | | N=20 | % | |
| Sex | Female | 11 | 55 | |
| | Male | 9 | 45 | |
| Bipolar diagnosis | I | 3 | 15 | |
| | II | 17 | 85 | |
| Age (year, mean ± SD) | | 23.95±7.44 | | |
| BMI (kg/m$^2$, mean ± SD) | | 21.91±3.56 | | |
| Onset age (year, mean ± SD) | | 19.75±6.75 | | |
| Duration of illness (year, mean ± SD) | | 4.25±3.71 | | |
| | | Pre-treatment | Post-treatment | P |
| MADRS score (mean ± SD) | | 25.75±7.87 | 11.29±8.35 | <0.001 |
| HDRS-17 score (mean ± SD) | | 28.25±7.27 | 11.65±8.76 | <0.001 |
| YMRS score (mean ± SD) | | 2.10±1.52 | 1.47±1.77 | 0.33 |
| Abbreviations: SD, standard deviation. | | | | |

**Table S4. 16S rRNA Sequencing Data from the Recruited Subjects**

| Group | Available_reads | Random_chose_reads | OBS | Shannon | simpson | invsimpson | obs | chao1 | ice | |
|---|---|---|---|---|---|---|---|---|---|---|
| BD | 46240 | 20000 | 212 | 3.02 | 0.88 | 8.45 | 181 | 203.13 | 189.73 | |
| BD | 32679 | 20000 | 192 | 2.27 | 0.76 | 4.08 | 163 | 217.38 | 179.85 | |
| BD | 58880 | 20000 | 58 | 1.4 | 0.6 | 2.51 | 36 | 56.17 | 52.31 | |
| BD | 65496 | 20000 | 184 | 3.32 | 0.93 | 14.29 | 143 | 173.38 | 152.39 | |
| BD | 39391 | 20000 | 113 | 1.96 | 0.72 | 3.6 | 92 | 144.56 | 105.66 | |
| BD | 59076 | 20000 | 178 | 2.16 | 0.73 | 3.72 | 127 | 165.23 | 137.1 | Discard |
| BD | 44069 | 20000 | 164 | 3.13 | 0.93 | 13.51 | 140 | 166.28 | 150.28 | |
| BD | 40081 | 20000 | 128 | 2.18 | 0.8 | 4.94 | 106 | 129.06 | 116.72 | |
| BD | 37355 | 20000 | 106 | 2.26 | 0.83 | 5.91 | 89 | 125 | 96.43 | |
| BD | 33767 | 20000 | 172 | 3.06 | 0.9 | 9.59 | 144 | 173.39 | 150.75 | |
| BD | 29266 | 20000 | 163 | 2.78 | 0.88 | 8.21 | 138 | 179.29 | 151.84 | |
| BD | 33406 | 20000 | 193 | 3.12 | 0.91 | 11.26 | 165 | 190.29 | 176.49 | |
| BD | 41989 | 20000 | 104 | 1.91 | 0.68 | 3.08 | 80 | 104.08 | 85.98 | Discard |
| BD | 52315 | 20000 | 204 | 1.35 | 0.38 | 1.62 | 158 | 188.86 | 170.86 | |
| BD | 49139 | 20000 | 125 | 1.39 | 0.48 | 1.93 | 93 | 122.39 | 101.18 | |
| BD | 38368 | 20000 | 127 | 1.97 | 0.64 | 2.8 | 112 | 153.14 | 120.55 | |
| BD | 37256 | 20000 | 157 | 2.64 | 0.84 | 6.12 | 138 | 168.73 | 146.92 | Discard |
| BD | 84800 | 20000 | 226 | 2.82 | 0.88 | 8.17 | 156 | 171.16 | 163.54 | |
| BD | 63966 | 20000 | 106 | 1.31 | 0.46 | 1.85 | 75 | 93.05 | 82.7 | |
| BD | 32100 | 20000 | 24 | 2.8 | 0.79 | 4.71 | 21 | 257. | 233. | |

| | | | | | | | | | | | |
|---|---|---|---|---|---|---|---|---|---|---|---|
| | | | 5 | | | | 5 | 88 | 89 | | |
| BD | 35515 | 20000 | 213 | 3.24 | 0.91 | 10.84 | 187 | 254.5 | 204.51 | | |
| BD | 25650 | 20000 | 127 | 2.99 | 0.9 | 10.46 | 107 | 149.67 | 111.86 | | |
| BD | 34465 | 20000 | 236 | 3.45 | 0.93 | 13.53 | 202 | 231.17 | 212.37 | | |
| BD | 44085 | 20000 | 125 | 2.46 | 0.84 | 6.27 | 99 | 113.45 | 104.78 | | |
| BD | 37447 | 20000 | 126 | 2.61 | 0.87 | 7.56 | 105 | 195 | 119.59 | | |
| BD | 38963 | 20000 | 206 | 2.3 | 0.68 | 3.12 | 170 | 200.12 | 181.68 | | |
| BD | 30276 | 20000 | 116 | 2.35 | 0.8 | 4.88 | 100 | 109.5 | 107.6 | | |
| BD | 30471 | 20000 | 198 | 2.13 | 0.65 | 2.88 | 167 | 201 | 180.66 | | |
| BD | 38880 | 20000 | 170 | 2.85 | 0.85 | 6.82 | 150 | 182.67 | 159.37 | Discard | |
| BD | 50290 | 20000 | 163 | 3 | 0.88 | 8.16 | 129 | 149.83 | 139.16 | | |
| BD | 31294 | 20000 | 157 | 3.03 | 0.91 | 10.87 | 137 | 150.33 | 143.47 | | |
| BD | 78146 | 20000 | 220 | 3.05 | 0.91 | 11.51 | 164 | 186.5 | 173.42 | Discard | |
| BD | 35979 | 20000 | 156 | 3.05 | 0.92 | 12.83 | 133 | 161.04 | 144.18 | | |
| BD | 46205 | 20000 | 284 | 2.95 | 0.8 | 5 | 225 | 262.5 | 240.08 | Discard | |
| BD | 43377 | 20000 | 159 | 2.76 | 0.87 | 7.92 | 126 | 168.25 | 134.58 | | |
| BD | 44736 | 20000 | 164 | 3.04 | 0.91 | 10.86 | 119 | 143.5 | 126.5 | | |
| BD | 37227 | 20000 | 145 | 2.96 | 0.9 | 9.6 | 124 | 173 | 134 | | |
| BD | 30284 | 20000 | 175 | 3.24 | 0.92 | 12.52 | 156 | 210 | 170.95 | | |
| BD | 39846 | 20000 | 118 | 2.64 | 0.87 | 7.79 | 98 | 131.06 | 106.03 | | |
| BD | 40227 | 20000 | 237 | 3.55 | 0.94 | 16.52 | 204 | 254.7 | 216.44 | | |
| BD | 31440 | 20000 | 164 | 2.91 | 0.85 | 6.69 | 149 | 207.91 | 163.07 | Discard | |

| | | | | | | | | | | |
|---|---|---|---|---|---|---|---|---|---|---|
| BD | 50906 | 20000 | 207 | 2.89 | 0.86 | 7.03 | 149 | 185.13 | 162.35 | Discard |
| BD | 33110 | 20000 | 179 | 3.34 | 0.93 | 13.86 | 156 | 202.72 | 165.41 | Discard |
| BD | 27298 | 20000 | 133 | 2.64 | 0.85 | 6.82 | 119 | 137.38 | 126.6 | |
| BD | 23899 | 20000 | 187 | 3.21 | 0.93 | 14.85 | 175 | 245.23 | 197.89 | Discard |
| BD | 43997 | 20000 | 312 | 3.74 | 0.94 | 17.69 | 250 | 280.42 | 262.08 | Discard |
| BD | 24871 | 20000 | 129 | 2.26 | 0.74 | 3.78 | 117 | 131.24 | 124.81 | Discard |
| BD | 29935 | 20000 | 155 | 2.8 | 0.88 | 8.11 | 139 | 191.07 | 147.71 | Discard |
| BD | 32283 | 20000 | 198 | 3.46 | 0.95 | 18.36 | 171 | 202.11 | 184.8 | |
| BD | 54836 | 20000 | 149 | 2.11 | 0.71 | 3.45 | 108 | 237.6 | 124.62 | Discard |
| BD | 22415 | 20000 | 123 | 2.09 | 0.76 | 4.25 | 114 | 144.15 | 125.51 | |
| BD | 39952 | 20000 | 178 | 2.7 | 0.81 | 5.32 | 146 | 194.05 | 156.51 | Discard |
| BD | 38105 | 20000 | 182 | 3.05 | 0.91 | 11 | 152 | 196.46 | 164.34 | |
| BD | 50314 | 20000 | 115 | 1.65 | 0.59 | 2.42 | 85 | 115.73 | 99.38 | |
| BD | 51022 | 20000 | 221 | 2.95 | 0.87 | 7.79 | 179 | 229 | 193.8 | |
| BD | 71955 | 20000 | 209 | 3.08 | 0.88 | 8.31 | 168 | 180.25 | 173.55 | |
| BD | 32870 | 20000 | 183 | 2.27 | 0.78 | 4.47 | 153 | 185.4 | 166.5 | Discard |
| BD | 26400 | 20000 | 144 | 2.79 | 0.88 | 8.38 | 125 | 155.73 | 133.8 | Discard |
| BD | 36953 | 20000 | 197 | 3.77 | 0.96 | 25.43 | 164 | 186.53 | 173.98 | |
| BD | 42349 | 20000 | 139 | 2.63 | 0.87 | 7.43 | 119 | 158.06 | 126.94 | |
| BD | 56395 | 20000 | 182 | 2.58 | 0.8 | 4.96 | 143 | 173 | 153.21 | |
| BD | 49871 | 20000 | 244 | 2.95 | 0.88 | 8.39 | 200 | 257.78 | 214.18 | |
| BD | 41342 | 20000 | 177 | 2.32 | 0.82 | 5.71 | 125 | 182.14 | 141.38 | Discard |

| | | | | | | | | | | |
|---|---|---|---|---|---|---|---|---|---|---|
| BD | 55649 | 20000 | 243 | 3.48 | 0.94 | 16.41 | 176 | 221.38 | 187.85 | Discard |
| BD | 69218 | 20000 | 216 | 2.75 | 0.84 | 6.19 | 154 | 192 | 167.44 | |
| BD | 50903 | 20000 | 132 | 1.37 | 0.49 | 1.97 | 89 | 118.39 | 96.49 | |
| BD | 55015 | 20000 | 186 | 2.72 | 0.87 | 7.87 | 144 | 180.3 | 156.86 | Discard |
| BD | 36867 | 20000 | 125 | 3.04 | 0.91 | 11.06 | 114 | 136 | 121.95 | |
| BD | 11735 | 11735 | 63 | 2.14 | 0.8 | 5.01 | 57 | 75 | 60.71 | |
| BD | 298358 | 20000 | 297 | 3.46 | 0.94 | 17.02 | 180 | 253.5 | 197.07 | |
| BD | 34784 | 20000 | 146 | 2.94 | 0.91 | 10.68 | 129 | 155.89 | 135.52 | |
| BD | 23110 | 20000 | 156 | 2.93 | 0.9 | 9.54 | 143 | 192.85 | 156.81 | |
| BD Treated | 27221 | 20000 | 188 | 2.58 | 0.79 | 4.67 | 173 | 209.03 | 186.18 | |
| BD Treated | 29797 | 20000 | 108 | 2.14 | 0.8 | 4.98 | 93 | 105.97 | 102.07 | |
| BD Treated | 38709 | 20000 | 160 | 0.75 | 0.21 | 1.26 | 128 | 229.25 | 147.51 | |
| BD Treated | 43087 | 20000 | 269 | 3.18 | 0.92 | 12.67 | 205 | 251.23 | 219.11 | |
| BD Treated | 35602 | 20000 | 144 | 2.84 | 0.88 | 8.2 | 118 | 129.64 | 122.84 | |
| BD Treated | 17999 | 17999 | 166 | 3.18 | 0.89 | 9.38 | 154 | 202.05 | 164.56 | |
| BD Treated | 13503 | 13503 | 191 | 1.98 | 0.55 | 2.21 | 180 | 200.64 | 190.65 | |
| BD Treated | 13910 | 13910 | 155 | 3.63 | 0.95 | 20.72 | 158 | 178.25 | 168.13 | |
| BD Treat | 63322 | 20000 | 122 | 1.98 | 0.78 | 4.58 | 87 | 113.18 | 96.23 | |

| | | | | | | | | | | |
|---|---|---|---|---|---|---|---|---|---|---|
| ed | | | | | | | | | | |
| BD Treated | 10991 | 10991 | 147 | 3.2 | 0.91 | 11.25 | 140 | 215 | 149.01 | |
| BD Treated | 64447 | 20000 | 176 | 3.02 | 0.9 | 10.19 | 123 | 158.04 | 134.74 | |
| BD Treated | 43105 | 20000 | 125 | 1.81 | 0.64 | 2.77 | 102 | 126.04 | 110.9 | |
| BD Treated | 45896 | 20000 | 179 | 2.63 | 0.77 | 4.43 | 141 | 155.29 | 147.37 | Discard |
| BD Treated | 19944 | 19944 | 160 | 2.77 | 0.85 | 6.59 | 151 | 183.03 | 163.43 | Discard |
| BD Treated | 25638 | 20000 | 161 | 3.16 | 0.93 | 13.71 | 147 | 208.25 | 159.93 | |
| BD Treated | 19409 | 19409 | 210 | 3.39 | 0.93 | 14.07 | 201 | 283.29 | 217.42 | |
| BD Treated | 12567 | 12567 | 73 | 2.39 | 0.82 | 5.49 | 69 | 125.25 | 74.73 | |
| BD Treated | 23198 | 20000 | 177 | 3.16 | 0.91 | 10.79 | 165 | 197.24 | 178.84 | Discard |
| BD Treated | 24480 | 20000 | 123 | 3.01 | 0.9 | 10.21 | 122 | 150.17 | 130.76 | |
| BD Treated | 64904 | 20000 | 168 | 2.61 | 0.87 | 7.83 | 126 | 177.57 | 142.53 | |
| BD Treated | 22179 | 20000 | 205 | 3.52 | 0.94 | 17.2 | 194 | 254.5 | 211.84 | |
| BD Treated | 24581 | 20000 | 160 | 3.37 | 0.94 | 15.85 | 142 | 170.8 | 148.75 | |
| BD Treated | 22480 | 20000 | 121 | 2.62 | 0.84 | 6.28 | 108 | 139.25 | 118.8 | |
| H | 39285 | 20000 | 14 | 2.83 | 0.87 | 7.83 | 12 | 171. | 136. | |

| | | | | | | | | | | |
|---|---|---|---|---|---|---|---|---|---|---|
| | | | 9 | | | | 9 | 25 | 75 | |
| H | 48572 | 20000 | 200 | 3.1 | 0.88 | 8.43 | 169 | 188.6 | 178.61 | |
| H | 51033 | 20000 | 200 | 3.33 | 0.92 | 12.35 | 172 | 195.36 | 182.63 | |
| H | 66009 | 20000 | 244 | 3.6 | 0.95 | 18.6 | 194 | 220.69 | 203.8 | |
| H | 47241 | 20000 | 270 | 3.79 | 0.95 | 21.13 | 229 | 281.36 | 246.35 | |
| H | 47266 | 20000 | 180 | 3.15 | 0.92 | 12.15 | 154 | 184.73 | 162.9 | |
| H | 40216 | 20000 | 197 | 3.19 | 0.91 | 11.19 | 168 | 187.53 | 175.41 | |
| H | 35014 | 20000 | 153 | 2.4 | 0.74 | 3.9 | 138 | 164 | 148.52 | |
| H | 37721 | 20000 | 109 | 1.19 | 0.39 | 1.63 | 86 | 161 | 100.46 | |
| H | 59331 | 20000 | 183 | 2.67 | 0.85 | 6.61 | 155 | 201.29 | 166.74 | |
| H | 24844 | 20000 | 191 | 3.3 | 0.92 | 13.21 | 174 | 212.03 | 192.56 | |
| H | 34017 | 20000 | 123 | 2.56 | 0.85 | 6.62 | 103 | 119.9 | 106.71 | |
| H | 54677 | 20000 | 130 | 1.72 | 0.69 | 3.21 | 97 | 130.8 | 106.62 | |
| H | 33348 | 20000 | 226 | 3 | 0.86 | 7.21 | 193 | 240.12 | 204.12 | |
| H | 34162 | 20000 | 70 | 1.54 | 0.6 | 2.48 | 55 | 71.67 | 58.02 | |
| H | 32607 | 20000 | 116 | 2.32 | 0.81 | 5.19 | 103 | 119.2 | 108.31 | |
| H | 35866 | 20000 | 295 | 3.67 | 0.95 | 19.42 | 219 | 267.40 | 233.09 | |
| H | 31549 | 20000 | 149 | 3.10 | 0.93 | 13.64 | 113 | 153.00 | 119.84 | |
| H | 33795 | 20000 | 355 | 4.00 | 0.96 | 25.92 | 275 | 337.50 | 291.45 | |
| H | 42037 | 20000 | 186 | 2.04 | 0.73 | 3.66 | 138 | 202.65 | 156.08 | |
| H | 39993 | 20000 | 277 | 3.07 | 0.86 | 6.90 | 202 | 263.54 | 214.82 | |
| H | 52848 | 20000 | 211 | 2.94 | 0.88 | 8.57 | 146 | 186.04 | 158.65 | |

| | | | | | | | | | | | |
|---|---|---|---|---|---|---|---|---|---|---|---|
| H | 45582 | 20000 | 224 | 2.20 | 0.71 | 3.49 | 164 | 218.86 | 182.90 | | |
| H | 37284 | 20000 | 275 | 2.51 | 0.72 | 3.55 | 209 | 230.03 | 217.83 | | |
| H | 46038 | 20000 | 207 | 2.24 | 0.81 | 5.26 | 154 | 186.40 | 165.74 | | |
| H | 33097 | 20000 | 235 | 2.69 | 0.84 | 6.41 | 186 | 234.09 | 203.32 | | |
| H | 32272 | 20000 | 243 | 2.06 | 0.64 | 2.77 | 182 | 213.13 | 196.46 | | |
| H | 13237 | 13237 | 240 | 2.49 | 0.70 | 3.29 | 208 | 246.35 | 221.56 | | |
| H | 39505 | 20000 | 286 | 3.27 | 0.90 | 10.07 | 218 | 258.02 | 231.70 | | |
| H | 28654 | 20000 | 294 | 3.89 | 0.96 | 27.62 | 228 | 266.03 | 240.44 | | |
| H | 32853 | 20000 | 273 | 3.69 | 0.96 | 23.12 | 212 | 240.44 | 221.66 | | |
| H | 40727 | 20000 | 258 | 2.85 | 0.87 | 7.46 | 190 | 218.66 | 199.37 | | |
| H | 31731 | 20000 | 280 | 3.57 | 0.94 | 16.57 | 224 | 308.96 | 239.75 | | |
| H | 32322 | 20000 | 205 | 2.87 | 0.86 | 7.08 | 154 | 182.04 | 163.50 | | |
| H | 31055 | 20000 | 266 | 3.13 | 0.90 | 9.79 | 211 | 267.25 | 227.98 | | |
| H | 30930 | 20000 | 216 | 3.68 | 0.96 | 23.32 | 165 | 189.00 | 172.66 | | |
| H | 28741 | 20000 | 255 | 3.27 | 0.91 | 10.90 | 200 | 248.66 | 215.06 | | |
| H | 22257 | 20000 | 264 | 3.82 | 0.95 | 21.58 | 212 | 293.00 | 222.76 | | |
| H | 42029 | 20000 | 286 | 3.16 | 0.92 | 12.73 | 195 | 237.47 | 206.12 | | |
| H | 24712 | 20000 | 299 | 3.41 | 0.92 | 12.00 | 236 | 280.08 | 252.19 | | |
| H | 31451 | 20000 | 373 | 3.92 | 0.96 | 22.84 | 294 | 372.77 | 316.62 | | |
| H | 27428 | 20000 | 122 | 2.53 | 0.88 | 8.07 | 92 | 124.40 | 97.92 | | |
| H | 63668 | 20000 | 417 | 3.11 | 0.85 | 6.56 | 273 | 327.39 | 291.72 | | |
| H | 37556 | 20000 | 251 | 2.03 | 0.64 | 2.79 | 191 | 239.02 | 208.02 | | |

| | | | | | | | | | | |
|---|---|---|---|---|---|---|---|---|---|---|
| H | 38333 | 20000 | 35<br>4 | 3.48 | 0.92 | 12.38 | 26<br>2 | 311.<br>47 | 283.<br>31 | |

**Table S5. Wicoxon rank sum test of principle coordinates in BD patients and healthy controls**

| PC | Cohort | P value |
|---|---|---|
| Unweighted_Unifrac_PC1 | BD_H | 0.004010234 |
| Unweighted_Unifrac_PC2 | BD_H | 0.006613568 |
| Unweighted_Unifrac_PC3 | BD_H | 0.000234761 |
| Unweighted_Unifrac_PC4 | BD_H | 0.015413256 |
| Unweighted_Unifrac_PC5 | BD_H | 0.00808057 |
| Unweighted_Unifrac_PC1 | BDtreated_H | 0.037808237 |
| Unweighted_Unifrac_PC2 | BDtreated_H | 0.005476419 |
| Unweighted_Unifrac_PC3 | BDtreated_H | 0.000253896 |
| Weighted_Unifrac_PC2 | BD_H | 8.56E-05 |
| Weighted_Unifrac_PC2 | BDtreated_H | 0.000918378 |
| Weighted_Unifrac_PC4 | BDtreated_H | 0.009204282 |
| Weighted_Unifrac_PC5 | BDtreated_H | 0.025863306 |
| Weighted_Unifrac_PC4 | BD_ BDtreated_ | 0.00752612 |
| Hellinger_PC1 | BD_H | 0.023938492 |
| Hellinger_PC2 | BD_H | 0.024349082 |
| Hellinger_PC3 | BD_H | 0.002706114 |
| Hellinger_PC4 | BD_H | 0.000968565 |
| Hellinger_PC4 | BDtreated_H | 0.023421163 |
| JSD_PC1 | BD_H | 0.016570556 |
| JSD_PC2 | BD_H | 0.015135017 |
| JSD_PC3 | BD_H | 0.0038417 |
| JSD_PC4 | BD_H | 0.00211161 |
| JSD_PC4 | BDtreated_H | 0.021901476 |
| Spearman_PC1 | BD_H | 0.00010472 |
| Spearman_PC3 | BD_H | 3.20E-08 |
| Spearman_PC4 | BD_H | 0.002017219 |
| Spearman_PC1 | BDtreated_H | 0.00199482 |
| Spearman_PC3 | BDtreated_H | 0.000226506 |
| Spearman_PC4 | BDtreated_H | 2.90E-05 |

| | | |
|---|---|---|
| Pearson_PC1 | BD_H | 0.024765569 |
| Pearson_PC2 | BD_H | 0.023938492 |
| Pearson_PC3 | BD_H | 0.000667811 |
| Pearson_PC5 | BD_H | 0.049912618 |
| Pearson_PC4 | BDtreated_H | 0.036668288 |
| Pearson_PC5 | BDtreated_H | 0.042655116 |

**Table S6. Genera to construct random forest models to distinguish BD and healthy controls**

| Genus | Mean (BD) | Mean (H) | P value | Enrich |
|---|---|---|---|---|
| Anaerofilum | 0 | 1.78E-05 | 0.001712543 | H |
| Brucella | 4.81E-06 | 0 | 0.030089686 | BD |
| Bacteroides | 0.417235558 | 0.282727841 | 0.001526887 | BD |
| Helicobacter | 0 | 4.44E-06 | 0.03078809 | H |
| Halomonas | 0.000103962 | 4.44E-05 | 0.000905155 | BD |
| Gemmiger | 0.006697115 | 0.011019461 | 0.009419587 | H |
| Roseburia | 0.026004924 | 0.049788418 | 0.016124731 | H |
| Parabacteroides | 0.022587608 | 0.01294098 | 0.000405006 | BD |
| Porphyromonas | 2.21E-05 | 1.00E-05 | 0.015499423 | BD |
| Escherichia/Shigella | 0.014265587 | 0.010825604 | 0.046620251 | BD |
| Delftia | 5.77E-06 | 0 | 0.030089686 | BD |
| Parasutterella | 0.009427885 | 0.013315036 | 0.011611481 | H |
| Leptotrichia | 9.62E-07 | 5.56E-06 | 0.031797739 | H |
| Coprococcus | 0.002905769 | 0.007006443 | 0.004615631 | H |
| UNCLASSIFIED_GENUS | 0.100295728 | 0.146505452 | 0.001456654 | H |
| Klebsiella | 0.00168677 | 0.00239279 | 0.040163271 | H |
| Slackia | 4.81E-06 | 2.00E-05 | 0.013171202 | H |
| Microcella | 6.73E-06 | 1.11E-06 | 0.039323546 | BD |
| Ruminococcus | 0.007025 | 0.013781164 | 0.01223063 | H |
| Rothia | 6.73E-06 | 3.11E-05 | 0.021262243 | H |
| Turicibacter | 0.000516694 | 7.39E-05 | 0.006286757 | BD |
| Anaerofustis | 5.77E-06 | 0 | 0.017217234 | BD |
| Peptoniphilus | 1.54E-05 | 3.33E-06 | 0.041360185 | BD |
| Sporobacter | 4.81E-06 | 1.33E-05 | 0.022840678 | H |
| Neisseria | 5.77E-06 | 1.83E-05 | 0.004260251 | H |
| Weissella | 6.06E-05 | 0 | 0.009885673 | BD |
| Erysipelotrichaceae_incertae_sedis | 0.001177502 | 0.001184444 | 0.040369485 | BD |
| Lachnospira | 1.77E-05 | 7.78E-06 | 0.034717402 | BD |
| Lactobacillus | 0.000204808 | 1.00E-05 | 0.000291309 | BD |

| | | | | |
|---|---|---|---|---|
| Faecalibacterium | 0.044677559 | 0.068554216 | 0.021408432 | H |

**Table S7. HDRS-17 and MADRS score changes following quetiapine treatment in BD patients**

| Number | HDRS-17 scores | | | MADRS scores | | |
|---|---|---|---|---|---|---|
| | Untreated | Treated | Reductive ratio | Untreated | Treated | |
| 1 | 29 | 7 | 0.76 | 23 | 9 | |
| 2 | 24 | 13 | 0.46 | 26 | 14 | |
| 3 | 33 | 34 | -0.03 | 25 | 26 | |
| 4 | 24 | 5 | 0.79 | 21 | 2 | |
| 5 | 28 | 5 | 0.82 | 25 | 5 | |
| 6 | 25 | 13 | 0.48 | 35 | 16 | |
| 7 | 35 | 12 | 0.66 | 41 | 7 | |
| 8 | 33 | 13 | 0.61 | 28 | 12 | |
| 9 | 18 | 8 | 0.56 | 16 | 9 | |
| 10 | 38 | NA | NA | 26 | NA | |
| 11 | 19 | 13 | 0.32 | 15 | 12 | |
| 12 | 35 | 10 | 0.71 | 28 | 9 | |
| 13 | 6 | 4 | 0.33 | 6 | 4 | Discard |
| 14 | 13 | 10 | 0.23 | 16 | 11 | Discard |
| 15 | 36 | 32 | 0.11 | 45 | 35 | |
| 16 | 24 | 10 | 0.58 | 20 | 8 | |
| 17 | 29 | 5 | 0.83 | 21 | 6 | |
| 18 | 22 | 18 | 0.18 | 26 | 19 | Discard |
| 19 | 40 | NA | NA | 26 | NA | |
| 20 | 18 | 9 | 0.50 | 17 | 11 | |
| 21 | 37 | NA | NA | 34 | NA | |
| 22 | 20 | 8 | 0.60 | 21 | 10 | |
| 23 | 20 | 1 | 0.95 | 22 | 1 | |

**Table S8. Genera to construct random forest models to distinguish quetiapine treatment response in BD patients**

| Genus | Mean (Respond) | Mean (Nonrespond) | P value | Enrich |
|---|---|---|---|---|
| Acinetobacter | 0 | 4.00E-05 | 0.015061822 | Nonrespond |
| Asaccharobacter | 0 | 6.00E-05 | 0.015223682 | Nonrespond |
| Eubacterium | 4.17E-06 | 0.00015 | 0.002262979 | Nonrespond |
| Paraprevotella | 0.003254669 | 0 | 0.007173648 | Respond |
| Lactococcus | 0 | 3.00E-05 | 0.015223682 | Nonrespond |
| Lachnospira | 3.90E-05 | 0 | 0.035646108 | Respond |
| Lactobacillus | 1.25E-05 | 0.00017 | 0.03624997 | Nonrespond |
| TM7_genera_incertae_sedis | 6.25E-05 | 0 | 0.036612569 | Respond |
| Achromobacter | 0 | 2.00E-05 | 0.015061822 | Nonrespond |
| Bifidobacterium | 0.0014375 | 0.01763 | 0.035603193 | Nonrespond |
